# A life-saving case of cardiopulmonary arrest with cardiac tamponade caused by erosion 6years after percutaneous atrial septal defect closure: a case report

**DOI:** 10.1186/s13019-021-01537-4

**Published:** 2021-05-21

**Authors:** Takuma Kobayashi, Taiji Watanabe, Haruka Fu, Okada Yohei, Tomoyuki Goto

**Affiliations:** 1 Cardiovascular Surgery, Japanese Red Cross Kyoto Daini Hospital, 355-5 Haruobi-cho, Kamanzadori, Marutamachi-agaru, Kamigyo-ku, Kyoto, 602-8026 Japan; 2 Department of Primary Care and Emergency Medicine, Graduate School of Medicine, Kyoto, Japan

**Keywords:** Atrial septal defect, Amplatzer septal occluder, Erosion

## Abstract

**Background:**

Cardiac erosion after percutaneous atrial septal defect (ASD) closure is a rare complication that requires immediate life-saving emergency surgery. In this report, we present our successful life-saving strategy for cardiac arrest due to cardiac tamponade caused by erosion 6years after the percutaneous closure of an ASD.

**Case presentation:**

The patient was a 50-year-old man who received treatment using an Amplatzer septal occluder (St. Jude Medical, St. Paul, MN, USA) treatment for ostium secundum atrial septal defect (size: 29.527.0mm) at another institution when he was 44years old.

**Conclusions:**

This case report presents a bailout surgical strategy for patients who are hemodynamically unstable with risks of coagulopathy and multiple organ failure. This case shows that cardiac surgeons need to be aware of percutaneous ASD-closure complications and should consider a bailout surgical strategy for patients at risk of multiple organ failure.

## Background

Amplatzer septal occluder (ASO) placement is a minimally invasive treatment for atrial septal defects. It is a safe procedure, and the mid-term and long-term outcomes have been reported as favorable [1]. Conversely, life-threatening complications due to ASO, such as erosion and cardiac tamponade, have been reported. These rare complications require immediate life-saving emergency surgery; however, no commonly accepted standard strategy for their treatment exists. Herein, we describe the successful treatment of cardiac arrest for cardiac tamponade due to erosion after treatment with ASO using a bailout surgical strategy.

## Case presentation

A 50-year-old man complained of sudden chest pain during exercise and subsequently collapsed due to cardiac arrest. Cardiopulmonary resuscitation was immediately started, and the patient was brought to the emergency department (ED). Six years prior to presentation, he had undergone successful ASO treatment using a 36-mm Amplatzer device at another hospital. No complications due to ASD had been reported. The defective foramen was 29.527.0mm^2^, the aortic rim was not deficient, and the superior rim was floppy. No problems occurred during the annual follow-ups.

**Fig. 1:**
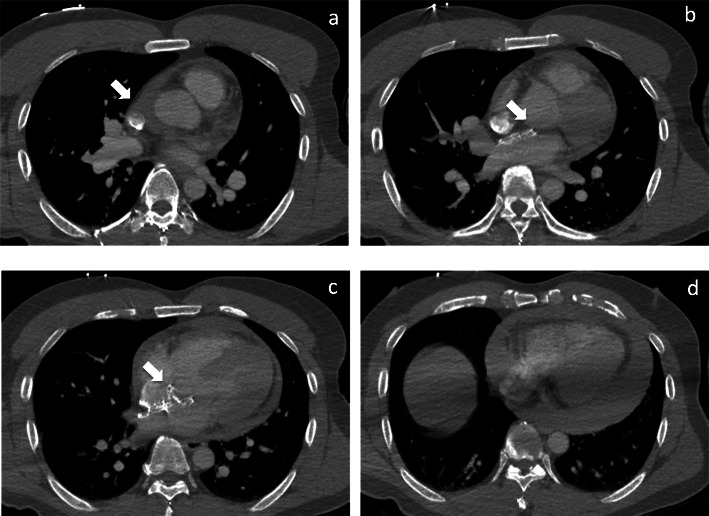
Computed tomography scan shows pericardial effusion with no evidence of aortic dissection. **a** Pericardial fluid accumulates near the transverse pericardial sinus, suggestive of erosion. **b**, **c** The edge of the Amplatzer septal occluder was in contact with the aortic wall from the anterior wall of the left atrium, adjacent to the aortic root. **d** The pericardial effusion was circumferential

Circulation spontaneously returned during transport. On arrival at the ED, he was intubated in a hypotensive condition with metabolic acidosis (lactate, 5.4mmol/L; HCO_3_,16.0mEq/L; base excess, 7.6mEq/L). Echocardiography showed pericardial effusion; contrast-enhanced computed tomography demonstrated no evidence of aortic dissection or extravasation of contrast medium from the myocardium or great vessels such as the aorta (Fig.1). Pericardial drainage was performed because he sustained a hemodynamically unstable status due to pericardial effusion. After 300mL of bloody effusion was drained, the patients condition improved substantially, but substantial blood drainage from the pericardial sac persisted. The patient was immediately transferred to the operating room, where surgical repair was performed.

**Fig. 2:**
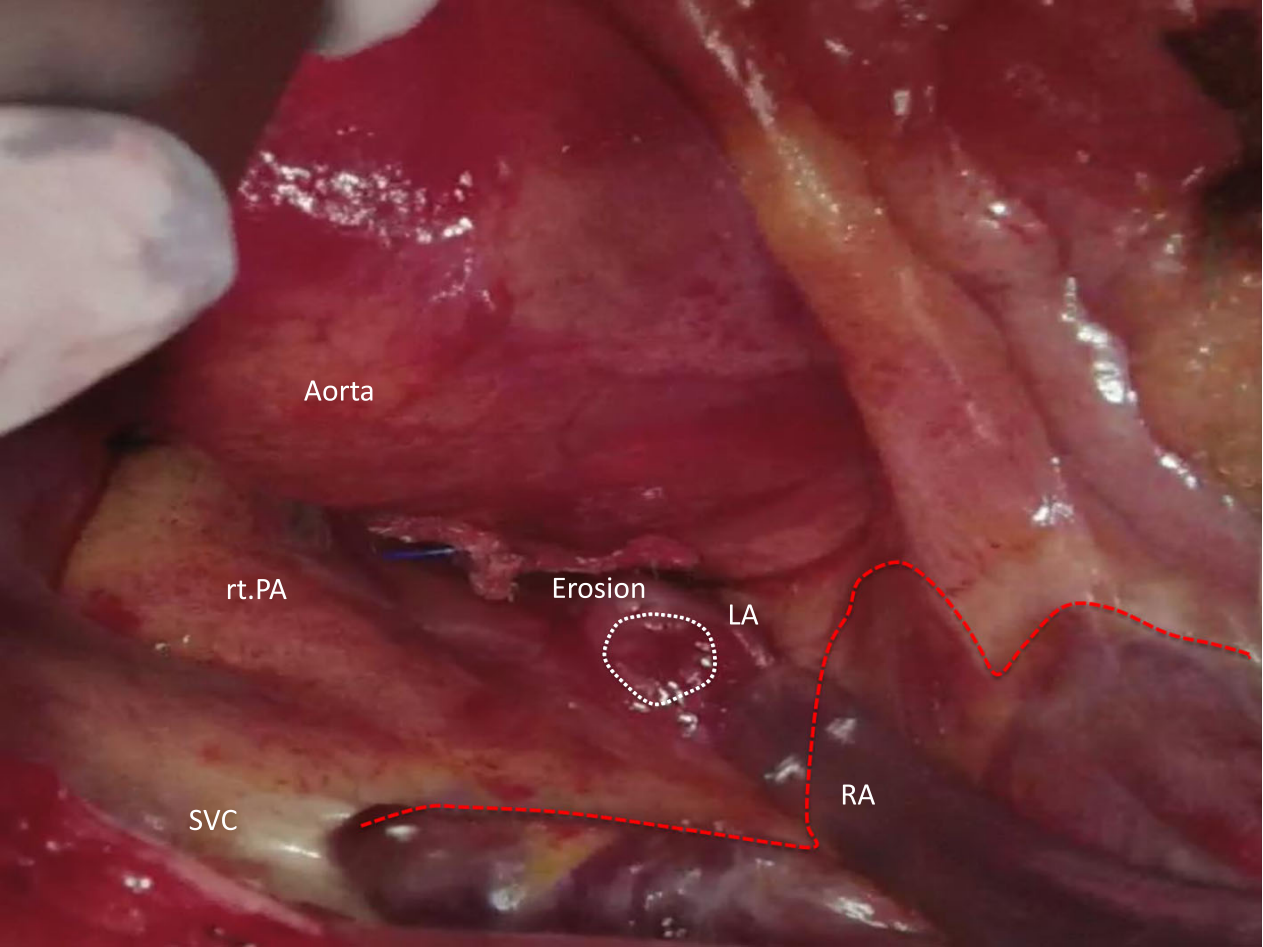
Operative view. A fistula was formed by the erosion in the area from the left atrium to the aortic root. There was no apparent extracardiac prolapse of the device. An autologous pericardial patch was formed during erosion to reinforce the left atrial wall. The aortic wall was sutured directly and repaired

The chest was opened through a midline sternal incision. While searching for the left atrial dome and the root of the aorta around the ASO device, the chord-like tissue had partially injured the left atrial dome, and hemorrhage was observed. Resection of the chordae revealed a fistula, which was diagnosed as ASO-induced erosion. There was no apparent extracardiac prolapse of the device, and an autologous pericardial patch was formed at the same site to reinforce the left atrial wall. The aortic wall was directly sutured and repaired (Fig.2). During the operation, hemodynamic improvement was observed with an increase in systemic blood pressure, and acidosis was normalized without cardiopulmonary bypass.

The patient was hemodynamically stable after admission to the intensive care unit. The patient was allowed to recover and wake up from anesthesia, and he was finally extubated without coma and had a good postoperative course. Postoperative contrast-enhanced computed tomography showed no evidence of extracardiac leakage of contrast medium or pericardial effusion due to persistent erosions. He was discharged from the hospital on day 12 post-surgery, walking unaided. Before hospital discharge, following adequate discussions with the interventional cardiologist and the patient, we decided to follow up with the previous surgeon who inserted the ASO. Four months after this surgery, the previous hospital reevaluated the erosion, and the device was removed. The erosion site and ASD were safely repaired in a planned cardiac surgery.

## Discussion and conclusions

This report describes the successful treatment of an out-of-hospital cardiac arrest case by immediate surgical repair for erosion after ASO without cardiopulmonary bypass. This case highlights that a bailout surgical strategy may be an important option for hemodynamically unstable patients with a risk of multiple organ failure, such as cardiac arrest patients.

This case has two important educational points. First, this case highlighted that ASD closure by the ASO carries a risk of life-threatening complications that cause cardiac tamponade and cardiac arrest. Cardiovascular surgeons should be aware of these rare complications and be prepared to perform immediate emergency surgery. Catheterization for ASD was first reported by Mills et al. [2]. In the late 1990s, the ASO was introduced and helped achieve better surgical outcomes and shorter hospitalization periods than surgical treatment. However, erosion of the cardiac wall caused by the device has been attracting attention in recent years as a rare complication that can cause rapid cardiac tamponade and can be fatal if appropriate measures are not taken. The majority of perforations occur within 48h after surgery, but the incidence of remote erosion is rare at 0.1% [3, 4]. Erosions occurring several months or years after implantation have also been reported [5,8], indicating that careful follow-up is essentialnot only during the acute phase but also for an extended period following surgery.

Second, we suggest that a bailout surgical strategy consisting of the immediate surgical control of bleeding without a cardiopulmonary bypass and planned reoperation for the permanent repair is an acceptable option to avoid multiple organ failure. Several strategies were previously proposed to resolve this complication. Amin et al. [3] reported that out of 28 cases of erosion, 21 patients underwent surgery (16 of which underwent device retrieval and closure of the perforation site, and 5 underwent closure of the perforation site only), and 7 underwent conservative treatment and observation. Although some studies have reported perforation repair performed without removing the device, other reports have advocated the prevention of recurrence by removal of the device and by careful follow-up [9]. Additional studies have indicated that emergency surgery for this complication has a high mortality rate. DiBardino et al. [10] reported that of 223 adverse events reported to the FDA, emergency surgery was performed in 152 cases, and 17 patients died, including 4 surgical deaths. This statistic shows that the mortality rate associated with emergency surgery for ASO-related complications is 20-times higher than that associated with conventional cardiac surgery.

In the present case, we avoided definitive surgery to remove the device and perform repair procedures using a cardiopulmonary bypass. We assumed that cardiopulmonary bypass could cause severe complications or multiple organ failure in cardiac arrest patients. Generally, cardiopulmonary bypass carries a risk of coagulopathy and organ failure [11]. Furthermore, cardiac arrest may cause organ injury such as rib fracture [12], hemothorax, and intra-abdominal bleeding caused by chest compression after resuscitation among these patients [13]. These injuries may lead to life-threatening major bleeding with coagulopathy. Moreover, following cardiac arrest, patients may develop a type of multiple organ failure called post-cardiac arrest syndrome caused by ischemic-reperfusion injury [14]. In this case, the possibility of further secondary injuries was not ruled out, and the progression to multiple organ failure due to cardiac arrest was considered. We therefore decided to close the perforation and reinforce the perforation site using autologous pericardium without extracorporeal hemodynamic support; we did not remove the ASO device. Therefore, it may be acceptable to perform temporary bleeding control for cardiac arrest patients to avoid a cardiopulmonary bypass.

Although reports of complications such as erosion are rare, the sudden occurrence of chest pain or pericardial effusion after ASO treatment should raise suspicion of erosion and prompt consideration of bailout surgical treatment as a damage control strategy.

We successfully treated a case of cardiac arrest due to cardiac tamponade caused by erosion 6years after the percutaneous closure of an atrial septal defect. We believe that the take-away point of this case is the importance of a bailout surgical strategy for patients who are hemodynamically unstable with risks of coagulopathy and multiple organ failure. This case suggests that cardiac surgeons need to be aware of the possible complications of percutaneous ASD closure and should formulate a bailout surgical strategy for patients at risk of multiple organ failure.

## Data Availability

The dataset supporting the conclusions of this article is included within the article, and any other inquiry is available from the corresponding author on reasonable request.

## Abbreviations

ASD: Atrial septal defect
ASO: Amplatzer septal occluder
ED: Emergency department

## References

[1] McElhinney DB, Quartermain MD, Kenny D, Alboliras E, Amin Z (2016). Relative risk factors for cardiac erosion following transcatheter closure of atrial septal defects a case-control study. Circulation..

[2] Mills NL, King TD (1976). Nonoperative closer of left-to-right shunts. J Thorac Cardio Vasc Surg.

[3] Amin Z, Hijazi ZM, Bass JL, Cheatham JP, Hellenbrand WE, Kleinman CS (2004). Erosion of Amplatzer septal occluder device after closure of secundum atrial septal defects: review of registry of complications and recommendations to minimize future risk. Catheter Cardiovasc Interv.

[4] Crawford GB, Brindis RG, Krucoff MW, Mansalis BP, Carroll JD (2012). Percutaneous atrial septal occluder devices and cardiac erosion: a review of the literature. Catheter Cardiovasc Interv.

[5] Enomoto N, Yasunaga H, Sakashita H, Shojima T, Todo K (2008). Late cardiac perforation after atrial septal defect closure with the Amplatzer septal Occluder. Jpn J Cardiovasc Surg.

[6] Taggart NW, Dearani JA, Hagler DJ (2011). Late erosion of an Amplatzer septal occluder device 6 years after placement. J Thorac Cardiovasc Surg.

[7] Tchantchaleishvili V, Melvin AL, Ling FS, Knight PA (2014). Late erosion of Amplatzer septal occluder device resulting in cardiac tamponade. Interact Cardiovasc Thorac Surg.

[8] Onakatomi Y, Asou T, Takeda Y, Ueda H, Goda M, Masuda M (2019). Aortic erosion occurring in over 5 years after Amplatzer septal Occluder implantation for secundum atrial septal defect: a case report. J Cardiothorac Surg.

[9] Preventza O, Sampath-Kumar S, Wasnick J, Gold JP (2004). Late cardiac atrial septal defect closure. Ann Thorac Surg.

[10] DiBardino DJ, McElhinney DB, Kaza AK, Mayer JE (2009). Analysis of the US Food and Drug Administration manufacturer and user facility device experience database for adverse events involving Amplatzer septal occluder devices and comparison with the Society of Thoracic Surgery congenital cardiac surgery database. J Thorac Cardiovasc Surg.

[11] Esper SA, Subramaniam K, Tanaka KA (2014). Pathophysiology of cardiopulmonary bypass: current strategies for the prevention and treatment of anemia, coagulopathy, and organ dysfunction. Semin Cardiothorac Vasc Anesth.

[12] Kralj E, Podbregar M, Kejar N, Balaic J (2015). Frequency and number of resuscitation related rib and sternum fractures are higher than generally considered. Resuscitation..

[13] Beom JH, You JS, Joung KM, Seung MK, Seok Y, Chung HS (2017). Investigation of complications secondary to chest compressions before and after the 2010 cardiopulmonary resuscitation guideline changes by using multi-detector computed tomography: a retrospective study. Scand J Trauma Resusc.

[14] Okada Y, Narumiya H, Kobayashi N, Hirotake N, Kotani H, Koike K (2019). Survival after cardiac arrest with instantaneous rigorlike stiffness: a case report. Ann Emerg Med.

